# Transfer learning improves performance in volumetric electron microscopy organelle segmentation across tissues

**DOI:** 10.1093/bioadv/vbaf021

**Published:** 2025-04-02

**Authors:** Ronald Xie, Ben Mulcahy, Ali Darbandi, Sagar Marwah, Fez Ali, Yuna Lee, Gunes Parlakgul, Gokhan S Hotamisligil, Bo Wang, Sonya MacParland, Mei Zhen, Gary D Bader

**Affiliations:** Terrence Donnelly Centre for Cellular & Biomolecular Research, University of Toronto, Toronto, ON, M5S 3E1, Canada; Peter Munk Cardiac Centre and Joint Department of Medical Imaging, University Health Network, Toronto, ON, M5G 2N2, Canada; Department of Molecular Genetics, University of Toronto, Toronto, ON, M5S 1A1, Canada; Vector Institute, Toronto, ON, M5G 0C6, Canada; Lunenfeld-Tanenbaum Research Institute, Mount Sinai Hospital, Toronto, ON, M5G 1X5, Canada; Lunenfeld-Tanenbaum Research Institute, Mount Sinai Hospital, Toronto, ON, M5G 1X5, Canada; Lunenfeld-Tanenbaum Research Institute, Mount Sinai Hospital, Toronto, ON, M5G 1X5, Canada; Terrence Donnelly Centre for Cellular & Biomolecular Research, University of Toronto, Toronto, ON, M5S 3E1, Canada; Terrence Donnelly Centre for Cellular & Biomolecular Research, University of Toronto, Toronto, ON, M5S 3E1, Canada; University of California, Berkeley, Berkeley, California, 94720, United States; Sabri Ülker Center of Metabolic Research and Department of Molecular Metabolism, Harvard T.H. Chan School of Public Health, Boston, MA, 02139, United States; Broad Institute of MIT and Harvard, Cambridge, MA, 02142, USA; Peter Munk Cardiac Centre and Joint Department of Medical Imaging, University Health Network, Toronto, ON, M5G 2N2, Canada; Vector Institute, Toronto, ON, M5G 0C6, Canada; Department of Laboratory Medicine and Pathobiology, Temerty Faculty of Medicine, University of Toronto, Toronto, M5S 3E1, Canada; Department of Computer Science, University of Toronto, Toronto, ON, M5S 3E1, Canada; Department of Laboratory Medicine and Pathobiology, Temerty Faculty of Medicine, University of Toronto, Toronto, M5S 3E1, Canada; Ajmera Transplant Centre, Toronto General Research Institute, University Health Network, Toronto, ON, M5G 2N2, Canada; Department of Molecular Genetics, University of Toronto, Toronto, ON, M5S 1A1, Canada; Lunenfeld-Tanenbaum Research Institute, Mount Sinai Hospital, Toronto, ON, M5G 1X5, Canada; Terrence Donnelly Centre for Cellular & Biomolecular Research, University of Toronto, Toronto, ON, M5S 3E1, Canada; Department of Molecular Genetics, University of Toronto, Toronto, ON, M5S 1A1, Canada; Lunenfeld-Tanenbaum Research Institute, Mount Sinai Hospital, Toronto, ON, M5G 1X5, Canada; Department of Computer Science, University of Toronto, Toronto, ON, M5S 3E1, Canada; Princess Margaret Cancer Centre, University Health Network, Toronto, ON, M5G 2C4, Canada

## Abstract

**Motivation:**

Volumetric electron microscopy (VEM) enables nanoscale resolution three-dimensional imaging of biological samples. Identification and labeling of organelles, cells, and other structures in the image volume is required for image interpretation, but manual labeling is extremely time-consuming. This can be automated using deep learning segmentation algorithms, but these traditionally require substantial manual annotation for training and typically these labeled datasets are unavailable for new samples.

**Results:**

We show that transfer learning can help address this challenge. By pretraining on VEM data from multiple mammalian tissues and organelle types and then fine-tuning on a target dataset, we segment multiple organelles at high performance, yet require a relatively small amount of new training data. We benchmark our method on three published VEM datasets and a new rat liver dataset we imaged over a 56×56×11μm volume measuring 7000×7000×219 px using serial block face scanning electron microscopy with corresponding manually labeled mitochondria and endoplasmic reticulum structures. We further benchmark our approach against the Segment Anything Model 2 and MitoNet in zero-shot, prompted, and fine-tuned settings.

**Availability and implementation:**

Our rat liver dataset’s raw image volume, manual ground truth annotation, and model predictions are freely shared at github.com/Xrioen/cross-tissue-transfer-learning-in-VEM.

## Introduction

Volumetric electron microscopy (VEM) generates nanoscale resolution images of cellular structures in three-dimensional (3D) at tissue scale, using serial two-dimensional (2D) sample sectioning. VEM has been widely used in the field of connectomics to study neuronal circuits in the brain (Arganda-Carreras *et al.* 2021, Witvliet *et al.* 2021, Peddie *et al.* 2022), with increasing use in other tissues, such as pancreas, liver, and bladder (Žerovnik Mekuč *et al.* 2020, Xu *et al.* 2021, Parlakgül *et al.* 2022).

Accurate segmentation of cellular structures in the captured image volumes is often the first step to downstream analysis (Kornfeld and Denk 2018). Traditionally, segmentation is performed manually, which is extremely laborious and requires expert knowledge. Furthermore, VEM data can be generated using several methods, including array tomography (Micheva and Smith 2007), FIB-scanning electron microscopy (SEM) (Heymann *et al.* 2006), and serial block face-SEM (Denk and Horstmann 2004), which produces images with different resolutions and other characteristics. The data are also prone to diverse artifacts, including misalignment of adjacent 2D images, brightness differences, and out of focus regions, which complicates segmentation (Borrett and Hughes 2016). These issues prompted the community to develop various methods to automate the segmentation process (Beier *et al.* 2017, Lee *et al.* 2017, Januszewski *et al.* 2018, Funke *et al.* 2019, Heinrich *et al.* 2021). The U-net architecture has shown excellent image segmentation performance applied to a wide variety of biological and medical image types (Ronneberger *et al.* 2015, Milletari *et al.* 2016, Isensee *et al.* 2021). U-nets have also been adapted to automate organelle segmentation in 3D VEM and have achieved excellent performance (Çiçek *et al.* 2016, Lee *et al.* 2017, Žerovnik Mekuč *et al.* 2020, Heinrich *et al.* 2021, Li *et al.* 2022). However, these models still require a large and diverse set of manual labels to train. Furthermore, these labels cannot be reused across image data from different tissues using traditional supervised machine learning (Liu *et al.* 2020), making it difficult to establish VEM with new cellular structure and tissue types. Transfer learning reuses neural network parameters trained on previously labeled data and then fine-tunes the learned features on a target task. As many features such as shapes, textures, and edges that can be learned in one object segmentation task are useful for a diverse range of segmentation tasks, pretraining data does not need to be the same cellular structure or tissue. Transfer learning has been successfully used to reduce manual label needs in a range of image analysis application areas (Deng *et al.* 2009), but extensive comparison between the transferability across different tissues or classes has not yet been studied in VEM.

We evaluate transfer learning performance by training a 3D U-net following the architecture of Lee *et al.* on multiple cellular structure label types [mitochondria, endoplasmic reticulum (ER), lipid droplets, and neurites] from existing urinary bladder (Žerovnik Mekuč *et al.* 2020), mouse cortex (Arganda-Carreras *et al*. 2021), and mouse liver (Parlakgül *et al.* 2022). VEM datasets as well as a new liver VEM dataset were generated. We show that transfer learning can substantially reduce the need for manual labeling of VEM data by reusing labels across datasets and segmentation tasks.

Lastly, we benchmark our approach against the Segment Anything Model 2 (SAM2) (Ravi *et al.* 2024) and MitoNet (Conrad and Narayan 2023). The SAM2 is a state-of-the-art promptable segmentation model pretrained on over 600 K annotated mask sequences from 51 K videos, as well as over 1 B masks from 11 M high-resolution images. These datasets are diverse, covering a variety of real-world scenes and various objects. However, the training data does not explicitly contain electron microscopy images. MitoNet on the other hand is exclusively trained using over 1.5 M electron microscopy images and more than 100 k mitochondria instances. In our experiments, both SAM2 and MitoNet were tested under either zero-shot setting, prompted (e.g. with bounding boxes and masks) and fine-tuned settings to assess their practical effectiveness in segmenting organelles such as mitochondria (M) and ER in our newly generated rat liver dataset.

We make available our model, as well as our rat VEM data and manually labeled mitochondria and ER masks covering a 3500×3500×36 px volume along with our manually corrected model predictions for the entire volume (56×56×11μm, 8 nm/pixel resolution, 219 serial 7000×7000 pixel images at 50 nm per section) as a community resource.

## Methods

### Rat liver sample preparation

A 4-month-old male rat weighing 180 g was sacrificed and perfused with 2% glutaraldehyde and 2% paraformaldehyde in 0.15 M sodium cacodylate buffer (pH7.4) through the left ventricle at a hydraulic pressure of 90 cm for 5 min. The liver was removed, cut into 2–5 mm chunks, and stored in the same fixative solution at 4°C until needed. Secondary fixation and staining were performed using a modification of the fBROPA method, without formalin (Genoud *et al.* 2018). Briefly, samples were incubated with 2% OsO 4 + 1.5% potassium ferrocyanide in 0.15 M sodium cacodylate buffer for 90 min at room temperature, switched to 1% OsO_4_ in 0.15 M sodium cacodylate buffer for 90 min, then washed with 0.1 M sodium cacodylate buffer. Samples were incubated with 320 mM pyrogallol in ddH_2_O for 30 min, washed with 0.15 M sodium cacodylate buffer, incubated with 40 mM OsO_4_ in distilled water for 90 min, and washed and stored in ddH_2_O overnight. The following day, they were stained in Walton’s lead aspartate for 60 min at 60°C, washed in ddH_2_O, dehydrated with an ethanol series followed by propylene oxide, then infiltrated with a graded series of Epon resin, and cured at 60°C for 24 h. Samples were trimmed and mounted on a stub that was transferred to a Gatan 3View stage inside a Zeiss Gemini SEM. Two hundred nineteen sections were imaged at a thickness of 50 nm, resolution of 8 nm/pixel, and tile dimensions of 7000×7000 pixels (56×56 µm).

Healthy male Dark Agouti rats were purchased from Envigo and bred under the Animal Research Center at the Ontario Cancer Institute in a specific pathogen-free facility. All experimental procedures followed principles and guidelines for the care and use of animals established by the Animal Resources Centre at the University Health Network and are in accordance with the guidelines of the Canadian Council of Animal Care. Rat experiments were performed at the Toronto General Research Institute, Toronto, ON, Canada, under the approval of the Institutional Committee on Animal Bioethics and Care (AUP 5840). All procedures were performed under isoflurane anesthesia, and all efforts were made to minimize suffering.

### Datasets and preprocessing

Table 1 describes the datasets used to benchmark transfer learning across domains.

**Table 1.** Data sources and their attributes.^a^

	SNEMI3D	Urocell	Mouse Liver	Rat Liver
Method	SEM	FIB-SEM	FIB-SEM	SBF-SEM
Organism	Mouse	Mouse	Mouse	Rat
Tissue	Cortex	Urothelium	Liver	Liver
Image Resolution (nm)	6×6×30	16×16×15	8×8×8	8×8×50
Rescaled Resolution (nm)	6×6×30	10.2×10.2×15	32×32×40	28×28×50
Train Size (# Patches)	3388	1728	2160	120
Test Size (# Patches)	484	432	2160	120
Labels	N	M	M, ER, LD	M, ER

a Datasets are normalized together by applying contrast limited adaptive histogram equalization (CLAHE) (Reza 2004) and Gaussian blur before rescaling to the appropriate resolution and patched into the input size of the 3D ResU-net (160×160×18). The labels N, M, ER, and LD stand for neurites, mitochondria, endoplasmic reticulum, and lipid droplet, respectively.

The SNEMI3D neurite dataset was downloaded from their challenge website (Arganda-Carreras *et al.* 2021). There are 100 images in total, with the first 72 used for training and the last 18 used for testing. Slices 73–82 were discarded to limit information leak between training and testing sets. The dataset is anisotropic with 6 nm XY resolution and 30 nm Z resolution.

The Urocell mitochondria dataset was downloaded from its GitHub repository (Žerovnik Mekuč *et al.* 2020). The dataset also contains Golgi and lysosome labels, but they were not used for our experiments because they are relatively sparse. The released data are already binned by a factor of three to produce a near isotropic dataset. However for our purposes, we upsampled the dataset by a factor of two in the X and Y dimensions to reproduce the anisotropic nature to match the other datasets used in our study, as we assumed this would facilitate transfer learning between datasets.

The mouse liver dataset was downloaded from EMPIAR (2023). The dataset contains 5638 serial images, each with an image size of 12 000×8000 pixels. We used ground truth labels for mitochondria, ER, and lipid droplets every fifth slice in the Z dimension to create anisotropicity in the dataset to match the SNEMI3D and the rat liver datasets. We also use only 180 such slices starting at slice 1000 and 2000, respectively, for the training and testing set to simulate data scarce scenario for which transfer learning is practically useful.

All datasets were processed using contrast limited adaptive histogram equalization (Reza 2004) before the pixel values were scaled and centered. The datasets were scaled accordingly to keep the size of the objects inside one patch consistent between tasks. The rescaled volumes were then tiled into 160×160×18 px patches before inputting into our neural network models.

### Training and evaluation

For our proposed method, the neural network architecture used was adapted from the best performing model of the SNEMI3D competition (web) (Supplementary Fig. S1). The input dimension of this network is 160×160×18. It consists of a downsampling and upsampling branch consisting of five layers with 28, 36, 48, 64, and 80 channels, respectively, and a 2D max pooling layer between each layer. Each layer consists of a 2D convolution followed by two 3D convolutions, with a residual connection between the first and third convolutional module. As the dataset is anisotropic, the max pooling was only performed in the XY direction, with the dimensions of Z remaining the same throughout. Furthermore, a single 5×5×1 convolution and corresponding transpose convolution is applied prior to the aforementioned convolutions in the first layer and before the output layer, respectively, which helps alleviate the anisotropic nature of the input volumes.

Our model was trained using the Dice and cross-entropy (DiceCE) loss and the ADAM optimizer (Kingma and Ba 2014) with an initial learning rate of 0.002. In preliminary work, we found that a unified DiceCE loss, which combines cross-entropy loss and Dice loss, is more robust across different domains and training tasks compared to either Dice or cross-entropy loss alone. Dice loss gave poorer performance than cross entropy when labels are balanced, whereas cross-entropy loss alone was less consistent in more imbalanced prediction tasks. Input patches are randomly subjected to flips, rotations, brightness, and contrast augmentations during training to increase the robustness of the model. Training was performed on a single V100 Nvidia graphics card and repeated for 100 epochs or until convergence.

For evaluation, we measure the intersection over union (IoU). Similar improvements are observed in terms of accuracy, positive predictive value, and true positive rate.

The SAM2 (Ravi *et al.* 2024) inference was run with default settings on the rat liver dataset raw images with no downsampling, using updated weights from sam2.1-hiera-small.pt. The zero-shot setting was performed by modifying the automatic mask generator example workflow, whereas the prompted setting was performed by adapting the image predictor example workflow.

The MitoNet (Conrad and Narayan 2023) model inference and fine-tuning were both performed using Napari (Napari Contributors 2019) by installing the Empanada (Empanada Developers 2024) package. Fine-tuning data was selected either via randomly placed points for the “32 patches” setting or via a grid of points spaced 256×256×1 pixels apart for the “All training masks” setting.

For visualization of the model’s segmentation predictions, we first binarize the pixel-wise predicted logits with a threshold of 0.5, followed by conversion into mesh via the marching cubes algorithm (Lorensen and Cline 1987). Finally, the resulting mesh is visualized using Blender (Blender Foundation 2022).

## Results

### Pretrained 3D ResU-net outperforms a baseline across varying amounts of target task training data

**Figure 1.**
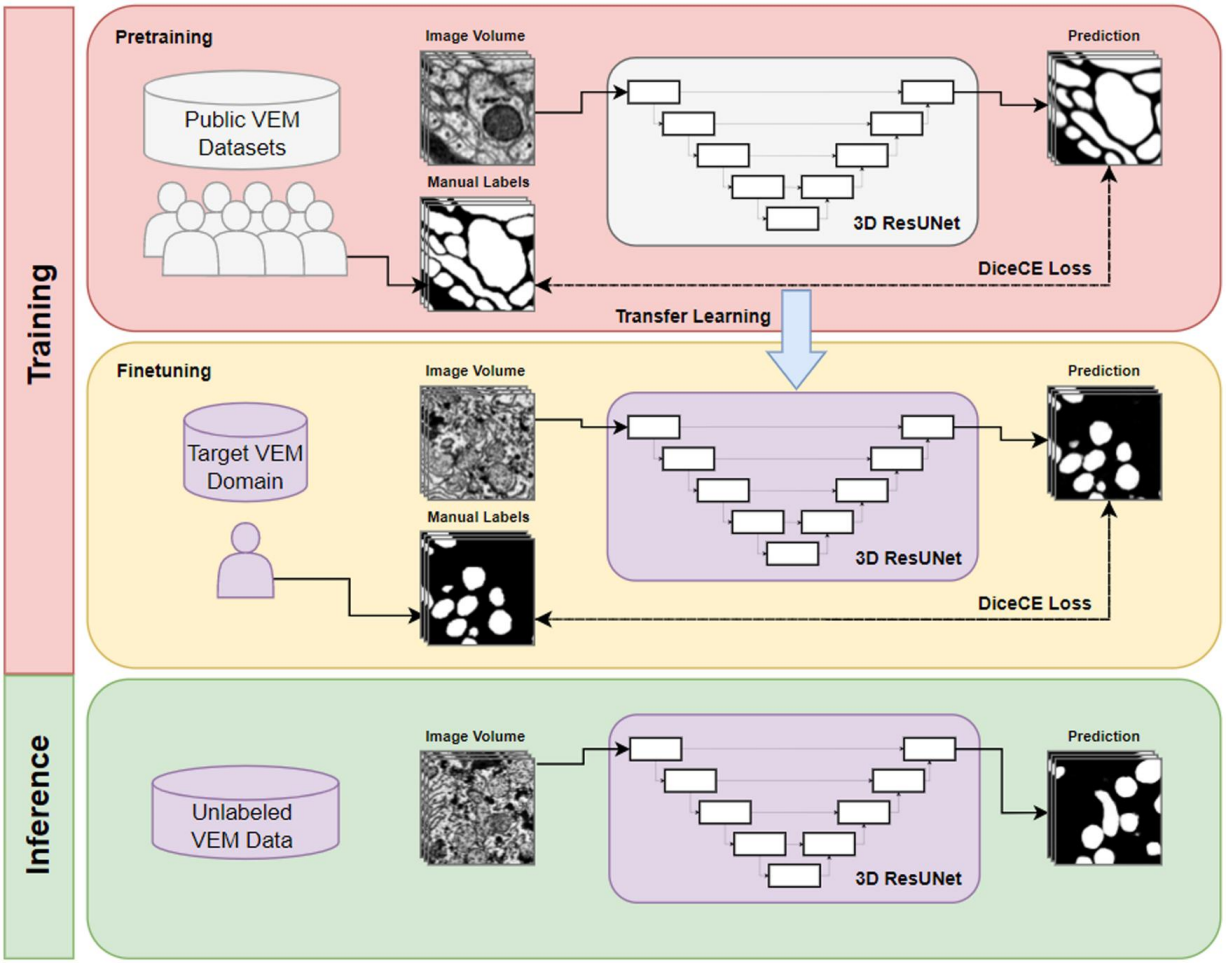
Transfer learning across domains. A randomly initialized deep learning model is first pretrained on a task with abundant labels from a different EM domain and subsequently fine-tuned on a target task where labels may be scarce. The transferred knowledge ideally alleviates the requirement for abundant labels for the target task and achieves better performance.

**Figure 2.**
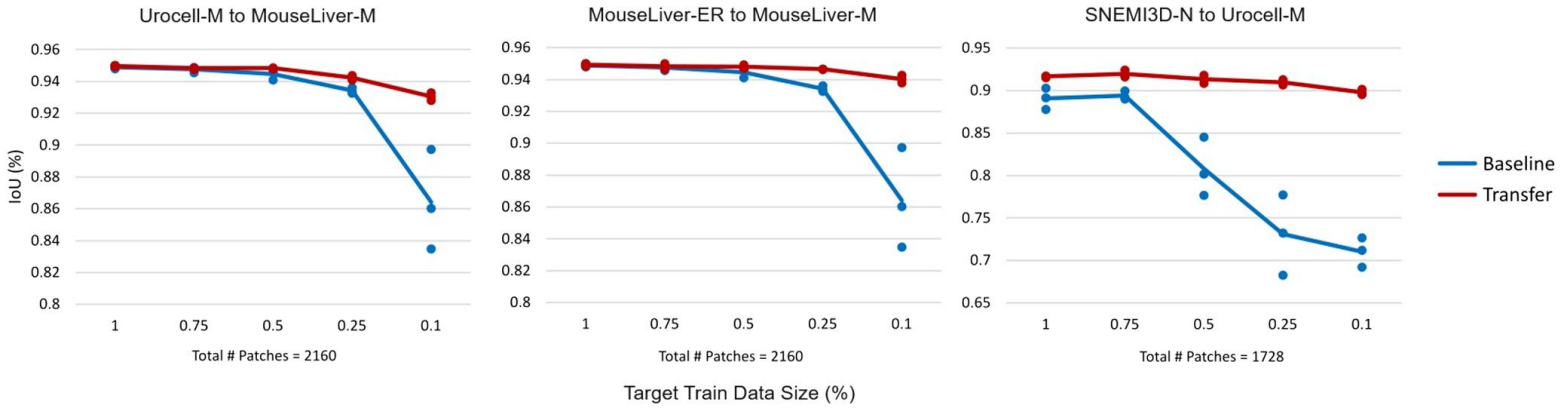
Transfer learning outperforms a random initialized baseline without transfer learning across three representative pretraining and target task scenarios. For each plot, “Baseline” values refer to the intersection-over-union (IoU) metric of the resulting segmentations from a model trained from scratch using randomly initialized weights compared to ground truth labels in a pixel-wise fashion, without using transfer learning. “Transfer” values refer to the IoU of the resulting segmentations from a model fine-tuned using the respective pretrained model compared to ground truth labels. For each pretraining and target task pair experiment, we vary the amount of training data available for the target task at five different levels (100%, 75%, 50%, 25%, 10%). The transferred networks surpass the performance of baseline particularly in training data scarce scenarios.

To implement transfer learning for VEM data, we pretrain a 3D ResU-net model from randomly initialized weights using images and segmentation labels from public data (Fig. 1). We then transfer the model by fine-tuning on a target task for a VEM image dataset of interest. Finally, the fine-tuned model is used to make predictions for the abundant unlabeled images in the target task. To measure the magnitude of the performance gain from transfer learning, we compare model performance with and without transfer learning and evaluate under three different pretraining and target task scenarios (Fig. 2): (i) the image volumes are from different tissues, but the objects being segmented are similar (both mitochondria segmentation), (ii) the image volumes are from the same tissue, but the objects being segmented are different (ER versus mitochondria segmentation), and (iii) the image volumes are from different tissues, and the objects being segmented are dissimilar (neurite segmentation versus mitochondria segmentation).

The amount of target training data used is varied at five different levels (100%, 75%, 50%, 25%, 10%) to simulate training in data scarce scenarios and to examine the extent transfer learning can help reduce manual labeling requirements for new datasets. Each experimental condition is repeated three times with either different randomly initialized weights (for the baseline) or different pretrained models from the same pretraining dataset (for transfer learning). We evaluate performance using IoU scores comparing predicted with known annotation pixels when the final models are applied to the target task test dataset. In all three cases, transfer learning consistently performs better than baseline on the target task, particularly when the amount of training data of the target task is low (Fig. 2).

**Figure 3.**
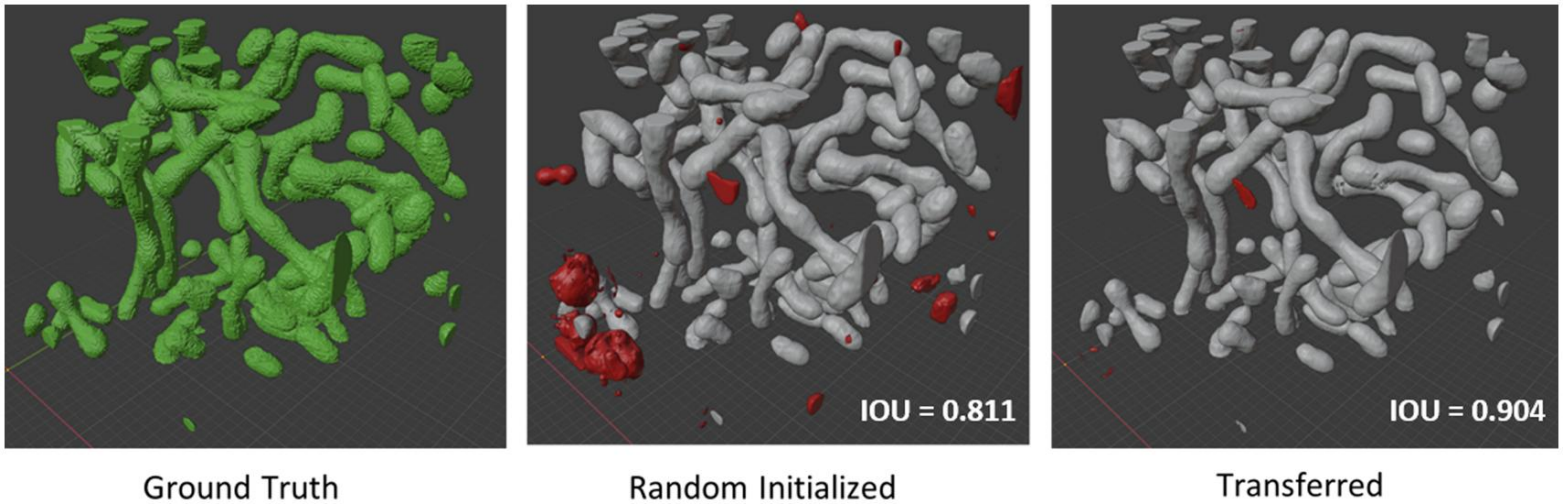
Transfer learning improves mitochondria segmentation in 3D. Binarized output of mitochondria segmentation model predictions. Large mispredictions (> 50% overlap) are colored in red. Both the randomly initialized model and the transferred model were trained with only 25% of training data. The transferred model makes noticeably fewer mispredictions.

As an additional test, we visualized the difference in the resulting segmentation of our 3D ResU-Net pretrained on the SNEMI3D-N (3D segmentation of neurites in EM Images) dataset versus randomly initialized baseline when only using 25% (432 patches) of the total available ground truth data for the Urocell mitochondria segmentation task (Fig. 3). The resulting IoU was 0.811 versus 0.904 for the randomly initialized and transferred models, respectively. Mispredictions with <50% overlap with ground truths are highlighted in red in Fig. 3. There are substantial false positives in the prediction of the randomly initialized model compared to minor mispredicted segments in the transferred model.

### Transfer learning confers substantial increase in performance across the majority of tested pretraining and fine-tuning task pairs

We next systematically evaluated how different pretraining and downstream task combinations affect the final performance of the resulting 3D ResU-Net (Table 2). Our results show that pretraining from VEM segmentation tasks in general boosts the performance of the target task, with the exception of one combination (Urocell-M transferred to MouseLiver-ER), regardless of how different the pretraining and target tasks are (Table 2). The highest average performance gain occurs with the mitochondria segmentation pretraining task from the mouse liver dataset (MouseLiver-M) with all target tasks (28% over random initialization).

**Table 2.** Transfer learning generally improves performance for different pretraining and fine-tuning task combinations.^a^

	SNEMI3D-N	Urocell-M	MouseLiver-M	MouseLiver-ER	MouseLiver-LD	RatLiver-M	RatLiver-ER
NoPretrain	60.68	65.71	60.84	66.35	52.75	76.81	73.14
SNEMI3D-N	80.81	88.24	92.54	72.23	80.43	**92.37**	76.04
Urocell-M	71.18	90.44	89.84	66.17	**82.83**	88.03	74.93
MouseLiver-M	**77.07**	**89.81**	94.82	**74.36**	81.80	92.08	76.43
MouseLiver-ER	76.60	87.15	**93.75**	78.02	67.62	92.04	**77.12**
MouseLiver-LD	69.73	85.00	89.52	67.46	84.72	87.76	75.01

a The 3D ResU-net is pretrained on a given dataset and task (rows). The pretrained model is then transferred and fine-tuned using 100 randomly selected patches from each target task (columns) for fine-tuning. The reported values in the table are the intersection over union (IoU) of predicted versus ground truth segmentations. The baseline values are randomly initialized deep learning models trained on the target task without transfer learning. Gray boxes represent the performance of the model when given 100% of the target task training data and serve as an upper bound comparison. Bolded values are combinations that produced the best performance for each target task-test pair.

### Transfer learning enables accurate mitochondria and ER segmentation of the rat liver dataset with limited task-specific ground truths

**Figure 4.**
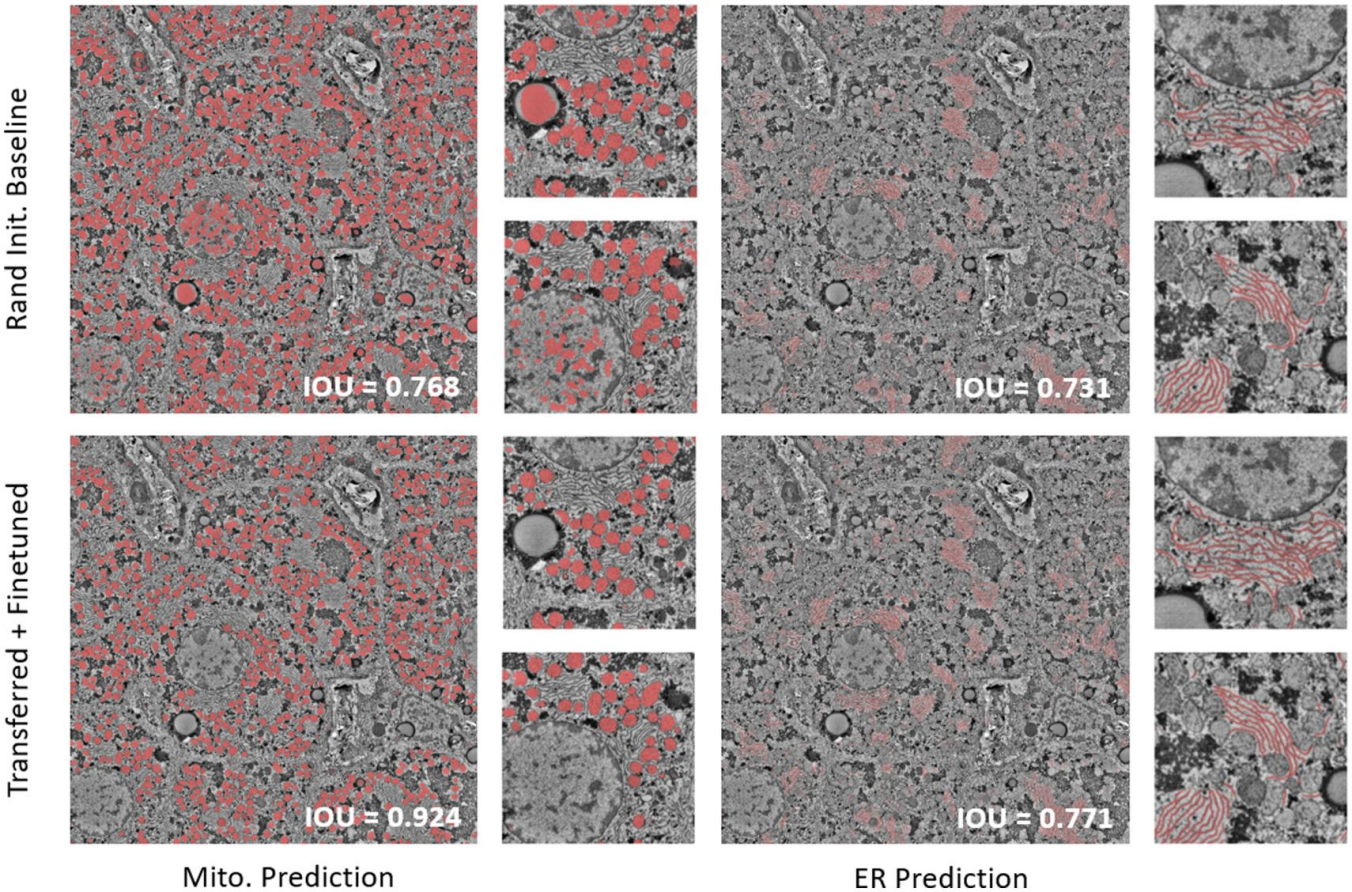
Transfer learning outperforms randomly initialized baseline for both mitochondria and ER prediction tasks in the rat liver dataset. Rand Init. Baseline: Rat liver dataset inference on previously unseen slice using a 3D ResU-Net with randomly initialized weights, trained on 18 slices of manual ground truths of mitochondria (left) and ER (right). Transferred + Fine-tuned: rat liver dataset inference on unseen slice using a model first pretrained on the SNEMI3D-N and MouseLiver-ER datasets, and then fine-tuned on the same 18 slices of manual segmentation masks for mitochondria (left) and ER (right), respectively. Red pixels indicate model predictions. Smaller image patches are zoom-ins of a region in the larger image to more clearly show the organelle structures. Zoom-ins are matched in the top and bottom figure sections. IoU values are shown for each image, with higher values being better.

**Figure 5.**
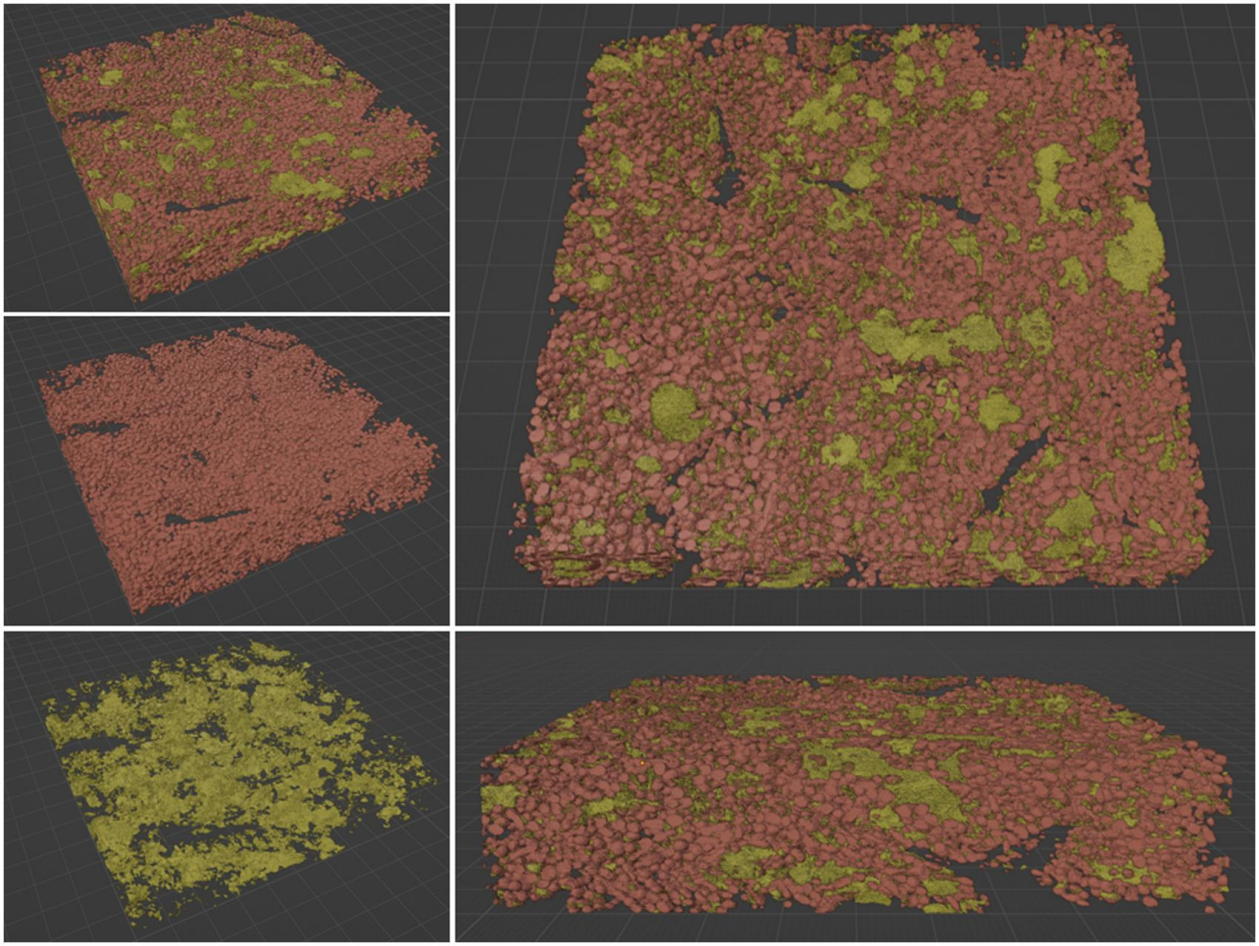
Automatic segmentation of mitochondria and ER of the entire rat liver volume using transfer learning and 3D ResU-Net. 3D ResU-Nets were pretrained using the best pretraining and target pair in Table 2, respectively, for mitochondria and ER segmentation of the rat liver dataset. The resulting model is used to infer annotation on all 219 serial sections of the dataset. The figures show the resulting 3D mesh of the ER and mitochondria predictions. Red pixels label mitochondria and yellow pixels label ER regions. Left: perspective view of the segmentation prediction for both mitochondria and ER (Top), mitochondria only (middle), and ER only (bottom). Right: top down (top) and side view (bottom) of the combined mitochondria and ER predictions on the entire rat liver volume.

Using the findings of Table 2, we chose the best performing pretraining task for the RatLiver-M and RatLiver-ER segmentation tasks, which are the SNEMI3D-N and MouseLiver-ER tasks, respectively, and fine-tuned on 120 patches of the corresponding task-specific ground truth labels that we manually annotated. As an additional test of our results on these data, we compared the fine-tuned and pretrained-only models to a model using both (transfer learning) for the RatLiver-M and RatLiver-ER tasks (Fig. 4) and confirmed by manual visual inspection that transfer learning did better than each approach separately. We applied this to our whole rat liver image (56×56×11μm volume, 8 nm/pixel resolution, 219 serial 7000×7000 pixel images at 50 nm per section) to automatically annotate mitochondria and ER segments (Fig. 5).

### Transfer learning outperforms several alternative approaches for mitochondria and ER segmentation of the rat liver dataset using limited task-specific ground truths

Table 3 benchmarks several existing practical alternatives to our described approach under zero-shot, prompted, and fine-tuned settings when labeling the rat liver dataset. As seen, existing state-of-the-art pretrained models, such as SAM2 (Ravi *et al.* 2024) and MitoNet (Conrad and Narayan 2023), both perform relatively poorly when generalizing to our rat liver dataset, despite being trained on billions of object masks in the case of SAM2, and in the case of MitoNet, on 1.5M electron microscopy images and more than 100 k mitochondria instances. Nevertheless, we show that the segmentation performance of these two models can be drastically increased with relatively low amounts of manual labor specific to the current task, which is important in practical settings where budget or time limitations permit only a small amount of the dataset to be annotated manually. We simulate such scenarios by either prompting models with bounding boxes and ground truth masks, or by fine-tuning models with manually generated ground truth segmentation masks in the rat liver training data.

**Table 3.** Comparison between different methods for organelle segmentation of the rat liver data.^a^

Method	Annotations	Organelle Class	IoU (%)
MitoNet 1×	None	M	25.11
MitoNet 2×	None	M	43.80
MitoNet 4×	None	M	5.66
MitoNet 1× fine-tuned	Training masks (32 patches)	M	74.21
MitoNet 2× fine-tuned	Training masks (32 patches)	M	87.44
MitoNet 1× fine-tuned	All training masks	M	87.37
MitoNet 2× fine-tuned	All training masks	M	89.11
SAM2	None	M	27.05
SAM2 + prompt	All bounding boxes	M	81.49
SAM2 + prompt	All bounding boxes	ER	22.24
SAM2 + prompt	All bounding boxes + masks	ER	21.46
Ours (best transferred + fine-tuned model)	Training masks (100 patches)	M	**92.37** (from Table 2)
Ours (best transferred + fine-tuned model)	Training masks (100 patches)	ER	**77.12** (from Table 2)

a The table reports the Intersection Over Union (IoU) achieved by various models under zero-shot, prompted or fine-tuned settings, and with different input upsampling (1×, 2×, 4×). The labels M and ER refer to mitochondria and endoplasmic reticulum, respectively. Highest performance for both M and ER segmentation are bolded.

We show that fine-tuning MitoNet using as little as 32 256×256 pixel patches can drastically improve its model performance over equivalent zero-shot settings. Also, pretraining with the entire training set generated for our rat liver dataset further improves the performance of the model on par with the performance of most pretraining and fine-tuning task combinations tried on our residual UNet model in Table 2.

Interestingly, for mitochondria, when SAM2 is provided with rough labels of bounding boxes of mitochondria, the performance already exceeds training a 3D resUNet model from a randomly initialized baseline (IoU = 81.49 versus 76.81).

For ER segmentation of our rat liver dataset, we did not find any appropriate models to perform zero-shot annotation. SAM2, e.g. had difficulty understanding the ER as an object, even in prompted settings where bounding boxes and rough segmentation masks are provided.

## Discussion

In this study, we investigated the benefit of transfer learning on organelle segmentation across 3D high-resolution VEM data taken from mouse cortex, mouse liver, mouse urinary bladder, and rat liver tissues. Accurate and scalable segmentation of these organelles is critical for downstream analysis for biological insights.

We show that transfer learning benefits segmentation in three different cases: when the pretraining task and the target task are trained from images from different domains but the segmentation task is similar; when the pretraining task and the target task are trained from images from the same domain but the segmentation task is different; and when the pretraining task and the target task are trained from images from different domains and the segmentation task is also different (Fig. 2). Furthermore, we showed that this performance increase becomes increasingly larger when the amount of training data available in the target task is reduced from 100% to 10%. These results show that there is generalizable knowledge across different VEM datasets. Similarly, there is generalizable knowledge across different segmentation tasks in these VEM datasets.

To manually annotate a 3500×3500×18px volume of images at 8×8×30 nm resolution for both mitochondria and ER would take roughly 100 h (based on our manual image annotation throughput), with time varying with the task difficulty, the density of the organelle in the particular field of view, the ability of the individual annotator, and image quality. Our results show promise to alleviate the time-consuming manual annotation bottleneck. Instead of manually annotating the whole image, as little as 10% of the image can be manually annotated, and then this can be used to fine-tune an existing pretrained model to predict the rest of the segmentation. This is especially important for applications of VEM in new tissue types, as there may be a lack of publicly available domain-specific datasets to train neural networks using standard approaches that do not use transfer learning.

We study this phenomenon by systematically evaluating the performance of models across different pretraining and fine-tuning task pairs, particularly including a newly acquired rat liver dataset with minimal manual labels as a practical scenario and proof of concept (Table 2). The target task is fine-tuned using 100 randomly selected patches of 160×160×18, to simulate a data-scarce scenario. This represents ∼18 h of annotation time extrapolated from our own experience with annotating the ER and mitochondria of the rat liver dataset. There are a few observations of interest. First, all pretraining and target task combinations outperform the randomly initialized baseline with the exception of one case where there is a small decrease in performance. This demonstrates the value of naively pretraining segmentation and annotation models on VEM datasets in general. This makes sense as concepts such as shape and edge detection are expected to be generally useful for segmentation of biological structures. Second, the MouseLiver-M pretraining task is consistently the best performing after transferring across all target tasks compared to other pretraining tasks. This may be because mitochondria segmentation provides a rich training signal in that it requires both good modeling of shape to distinguish from other non-rotund objects and structures, as well as good modeling of texture and edges to distinguish between rotund objects as well as between ER and mitochondria cristae. Third, for the rat liver dataset, the SNEMI3D-N pretraining task somewhat outperformed MouseLiver-M. This is rather unexpected as the SNEMI3D-N data (mouse brain, neurites) are both cross-domain and cross-task compared to the rat liver dataset. This may be because the SNEMI3D-N neurite segmentation task visually resembles the shape and size of mitochondria and contains potentially more variability for better generalization. This result highlights the potential of using relatively widely available connectomics VEM datasets to improve training of less widely available non-connectomics-related segmentation tasks. In contrast, the MouseLiver-ER pretraining task confers the most benefit on the RatLiver-ER segmentation task, likely due to task similarity between the pair. This shows that while pretraining on VEM datasets in general benefits the performance substantially, it can still be beneficial for the pretraining task to be more similar to the target task.

We benchmarked our described pretraining + fine-tuning approach against SAM2 (Ravi *et al.* 2024) and MitoNet (Conrad and Narayan 2023), two state-of-the art alternatives for labeling our newly generated rat liver dataset. As seen in Table 3, while these two models have been trained on billions of object masks in the case of SAM2, and in the case of MitoNet, on 1.5 M electron microscopy images and more than 100 k mitochondria instances, their performance in zero-shot setting remains relatively poor. This highlights an important but practical issue of generalizability of existing solutions for segmenting newly generated datasets that may be out of distribution to ones used to pretrain the models.

Notably, the segmentation performance can drastically increase with a relatively low amount of manual labor specific to the current task by either generating small amounts of manually generated segmentation masks for fine-tuning or by creating rough bounding boxes for the objects to be segmented. We show that by fine-tuning MitoNet on as little as 32 256×256 pixel patches of manually generated segmentation masks for mitochondria drastically increases its performance on the testing set for our rat liver dataset (IoU 43.80 versus 87.44). This shows that transfer learning is quite agnostic to the model and confers benefit in a generalizable way. While this may not be a surprising finding by itself, it helps highlight that pretraining on a large and diverse corpus of datasets, both in the case of SAM2 and MitoNet, may be unnecessary for achieving good labeling performance in a fine-tuned setting, especially as such pretraining is resource intensive. The small amount of task-specific labels is practical to generate and is often sufficient to allow the fine-tuned models to generalize to the task. Furthermore, fine-tuning on the entire training dataset with MitoNet still does not outperform our pretrained + fine-tuned residual UNet model under the best tested task pair combinations.

We hypothesize that certain datasets are more compatible for generalizing to a specific downstream task than others during pretraining. When pretraining on a diverse mix of these datasets, the resulting model will benefit from those datasets while also being potentially negatively influenced by other datasets that may not be as helpful. If the computational budget allows, it may be worthwhile to individually evaluate the impact of certain pretraining datasets for their benefit provided to the downstream task and train the best performing model from the most suitable datasets in this way. Indeed, we observe substantial fluctuations across different pretraining + fine-tuning task pairs as seen in Table 2, highlighting the importance of such compatibility across datasets.

We also briefly evaluated alternative ways of using limited manual efforts for dataset annotation in the form of generating bounding boxes rather than complete masks, the former being much faster to generate. Interestingly, for mitochondria, when SAM2 is provided with rough labels of bounding boxes, the resulting segmentation performance already exceeds training our residual UNet model from randomly initialized weights using manual labels. This highlights that investing in generating bounding boxes for objects of interest rather than precise masks for a new dataset is a viable alternative for annotating the dataset, potentially saving costs and time by eliminating the need to generate precise masks for these objects.

Nevertheless, prompting a segmentation model in this way still produces inferior results compared to fine-tuning using precise human-generated masks. This is especially true when the class relatively does not exhibit much variability across slices, such as the case for mitochondria in our dataset, making providing detailed annotations of just a few examples better than providing rough annotations (bounding boxes) of all objects. However, of note is that the performance of such an approach may be evaluated unfairly as it is affected by the inter-annotator variability of the ground truths the model predictions are benchmarked on, which a fine-tuned model will unfairly have access to by training on those annotations.

Overall, our experiments highlight the promise of using transfer learning to improve automatic segmentation performance across diverse organelles and tissue types. Transfer learning is particularly effective in data scarce scenarios, demonstrating its utility in emerging application areas of VEM. We also release our model and newly collected rat liver data and its accompanying manual labels as a resource for the community to further facilitate the adaptation and encourage the wider use of VEM in new biological domains.

## Supplementary Material

vbaf021_Supplementary_Data
