# Supplementary figures and images for: Transfer learning improves performance in volumetric electron microscopy organelle segmentation across tissues
Source: Bioinform Adv. 2025 Apr 2;5(1):vbaf021. doi: 10.1093/bioadv/vbaf021 (PMC11974384; doi:10.1093/bioadv/vbaf021)

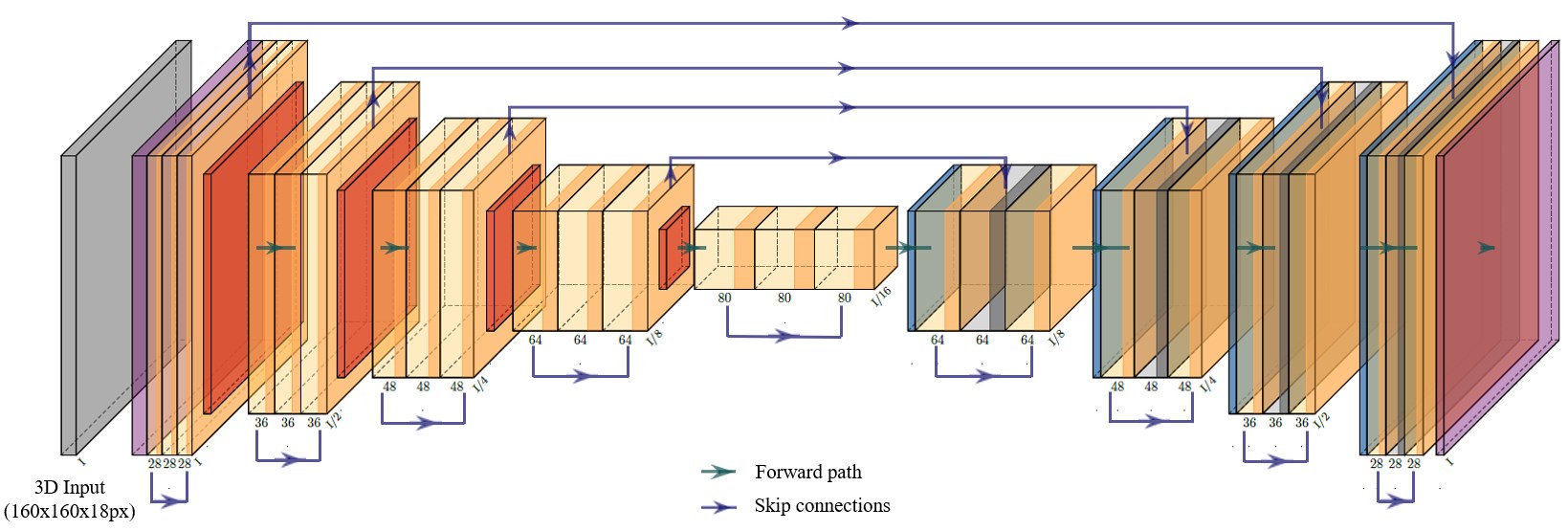

Supplement: vbaf021_Supplementary_Data [file vbaf021_supplementary_data.jpeg]
